# Pleomorphic Structures in Human Blood Are Red Blood Cell-Derived Microparticles, Not Bacteria

**DOI:** 10.1371/journal.pone.0163582

**Published:** 2016-10-19

**Authors:** Adam J. Mitchell, Warren D. Gray, Max Schroeder, Hong Yi, Jeannette V. Taylor, Rebecca S. Dillard, Zunlong Ke, Elizabeth R. Wright, David Stephens, John D. Roback, Charles D. Searles

**Affiliations:** 1 Division of Cardiology, Department of Medicine, Emory University, Atlanta, Georgia, United States of America; 2 Division of Infectious Disease, Department of Medicine, Emory University, Atlanta, Georgia, United States of America; 3 Robert P. Apkarian Integrated Electron Microscopy Core, Emory University, Atlanta, Georgia, United States of America; 4 Division of Infectious Disease, Department of Pediatrics, Emory University, Atlanta, Georgia, United States of America; 5 School of Biology, Georgia Institute of Technology, Atlanta, Georgia, United States of America; 6 Center for Transfusion and Cellular Therapy, Department of Pathology and Laboratory Medicine, Emory University, Atlanta, Georgia, United States of America; 7 Section of Cardiology, Atlanta VA Medical Center, Decatur, Georgia, United States of America; Massachusetts Institute of Technology, UNITED STATES

## Abstract

**Background:**

Red blood cell (RBC) transfusions are a common, life-saving therapy for many patients, but they have also been associated with poor clinical outcomes. We identified unusual, pleomorphic structures in human RBC transfusion units by negative-stain electron microscopy that appeared identical to those previously reported to be bacteria in healthy human blood samples. The presence of viable, replicating bacteria in stored blood could explain poor outcomes in transfusion recipients and have major implications for transfusion medicine. Here, we investigated the possibility that these structures were bacteria.

**Results:**

Flow cytometry, miRNA analysis, protein analysis, and additional electron microscopy studies strongly indicated that the pleomorphic structures in the supernatant of stored RBCs were RBC-derived microparticles (RMPs). Bacterial 16S rDNA PCR amplified from these samples were sequenced and was found to be highly similar to species that are known to commonly contaminate laboratory reagents.

**Conclusions:**

These studies suggest that pleomorphic structures identified in human blood are RMPs and not bacteria, and they provide an example in which laboratory contaminants may can mislead investigators.

## Background

Red blood cell (RBC) transfusions are a common and often life-saving therapy, but have been associated with significant morbidity and mortality[1]. The mechanisms responsible for this association remain unclear. During the course of studying RBC-derived microparticles (RMPs), which originate from RBC membrane blebbing and accumulate over time in stored human RBC units, we detected submicron, pleomorphic structures by negative-stain electron microscopy (EM). A review of the literature revealed previous reports of identical, submicron, pleomorphic structures in human blood that were characterized as bacteria [2, 3]. McLaughlin et al. concluded that the pleomorphic structures were bacteria based on bacterial 16S rDNA sequencing, flow cytometry-based fluorescent *in situ* hybridization studies, the apparent ability of the structures to replicate, and their sensitivity to antibiotics [2]. However, bacteria could not be cultured by standard techniques. Intrigued by the possibility of viable nanobacteria in RBC transfusion units as a possible etiology of poor clinical outcomes after transfusion, we examined the pleomorphic structures isolated from RBC storage units further, and conclude that these structures are not bacteria, but rather RMPs.

## Results

### Electron microscopy of RMPs

**Fig 1:**
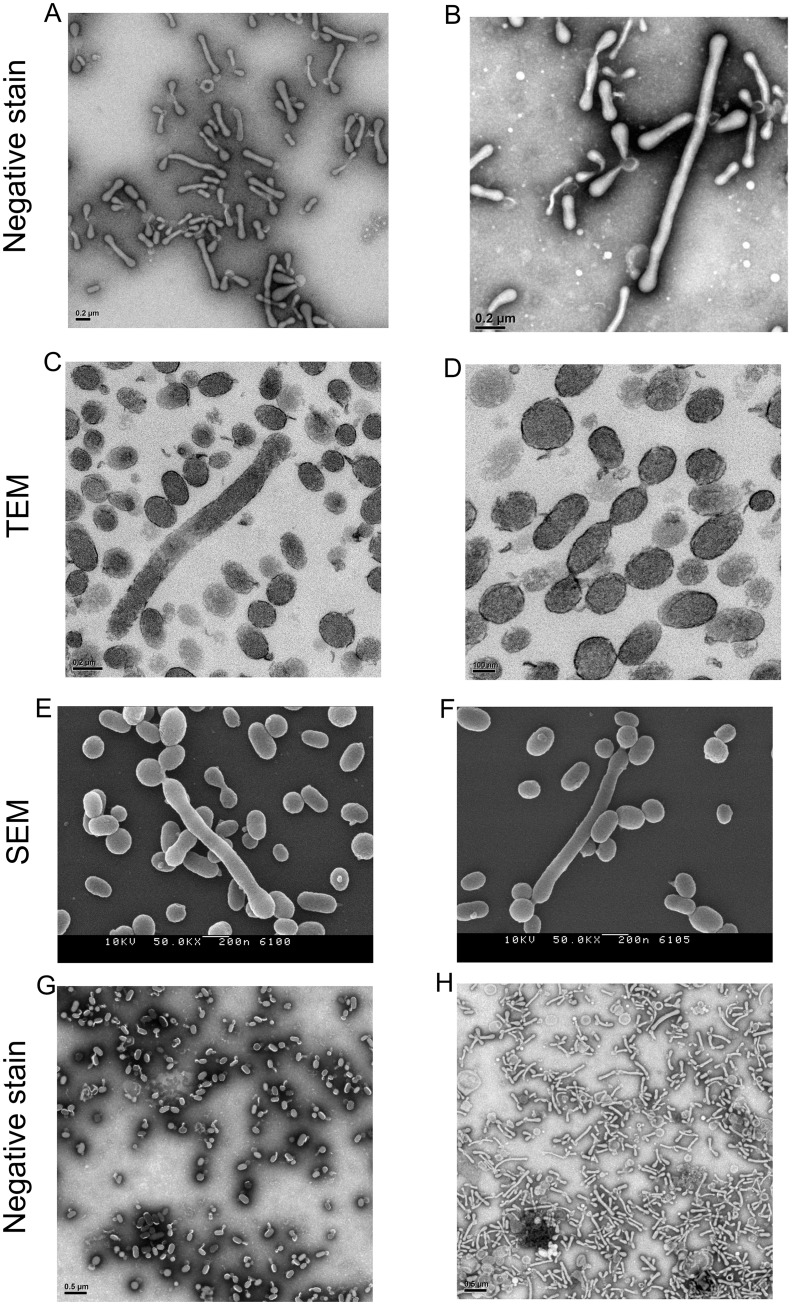
Representative electron micrographs of pelleted material from supernatant of RBC storage units. **A, B,** Negative-stain EM images of the unfixed material, showing pleomorphic structures. **C,D,** Thin-section TEM images, showing membrane encapsulate vesicles. **E,F,** SEM images. **G,H,** Negative-stain EM of fixed (G) versus unfixed (H) structures. Vesicles were isolated and imaged as described in Methods section.

**Fig 2:**
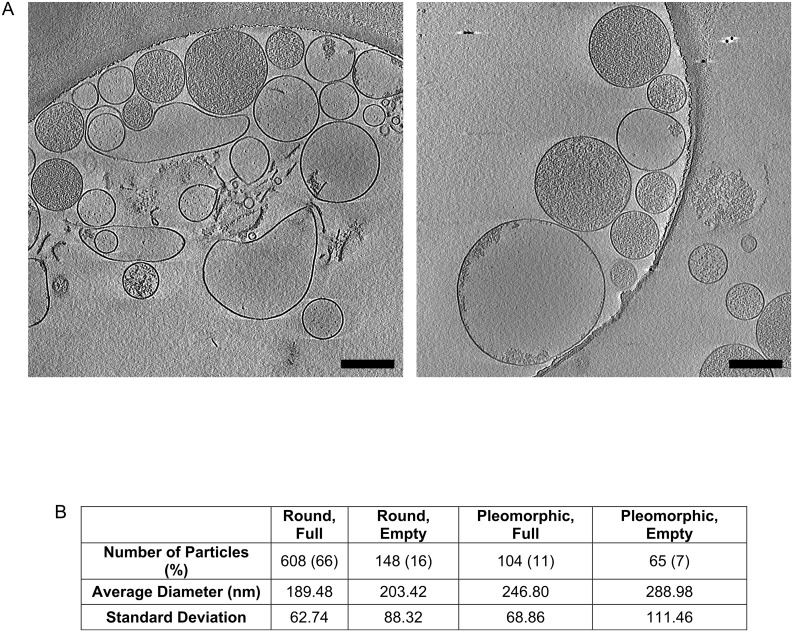
Representative 3D cryo-electron tomograpahy data and visual characterization of RBC-derived microparticles (RMPs). **A,** Slices through 3D volumes of RMPs. **B,** RMPs were measured and were characterized as either round or pleiomorphic, and as either full or empty. The number of each particle type, percent of the total, mean diameter, and standard deviation are presented in the table.

Several groups have published electron micrographs of RMPs, thus we expected to find a mostly spherical morphology [4, 5]. However, negative-stain EM images (Fig 1A and 1B) of the unfixed pellet obtained from the supernatant of stored RBC units appeared identical to images published by McLaughlin et al. and Szymanski et al.[2, 3]. In both of these latter cases, the pleomorphic structures were reported to be bacteria. We consistently identified similar pleomorphic structures in RBC units obtained from >6 healthy donors; these structures were present immediately after donation (day 0) as well as after several weeks of storage at 4°C, under standard blood bank conditions. We reasoned that these pleomorphic structures were either RMPs with unusual morphology (possibly due to artifact), or they were in fact microbial in nature. We systematically ruled out possible sources of artifact, including: RBC centrifugation, washing of the carbon grid with water, a lack of albumin in the isolated pellet. We also performed TEM and SEM of fixed pellets (Fig 1C–1F). Regardless of how the unfixed samples were prepared, negative-stain EM reliably produced images represented in Fig 1A and 1B. However, detailed analysis of fixed material by TEM and SEM revealed that, whereas some of the vesicles were pleomorphic and rod-like in shape, many retained the expected ellipsoidal shape (Fig 1C–1F) [4, 5]. Further, comparison of fixed and unfixed samples by negative-stain EM showed mostly ellipsoidal versus mostly pleomorphic, rod-like shaped vesicles respectively (Fig 1G and 1H). To definitely determine the native structure of the vesicles, two-dimensional (2D) cryo-electron microscopy (cryo-EM) images were acquired and three-dimensional (3D) cryo-electron tomography (cryo-ET) data sets were recorded and processed. 2D Cryo-EM micrographs and 3D cryo-ET reconstructions revealed predominantly round (77%) rather the pleomorphic vesicles (23%) and the presence of a single lipid bilayer (Fig 2) instead of the complex cell wall of a bacterial species. Average vesicle diameter was 190–288 nanometers. Interestingly, while most vesicles appeared to be filled with a dense granular substance, a significant fraction (23%) did not, appearing “empty” (Fig 2A and 2B).

### Microbial DNA analysis of vesicles identifies common contaminants

**Fig 3:**
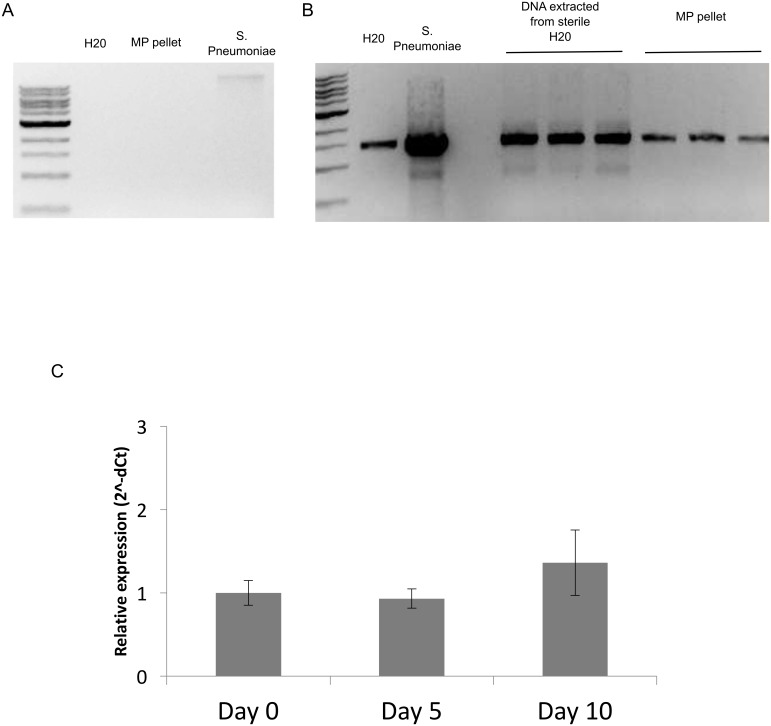
Analysis of bacterial DNA in pelleted material from RBC storage units and serum. RMPs were isolated from ~100 mL of RBCs stored under standard conditions for 42 days. **A**, Genomic DNA was isolated and analyzed by gel electrophoresis. Molecular grade water and DNA from *S*. *Pneumoniae* served as negative and positive controls, respectively. Band reflective of genomic DNA was only observed in positive control lane. **B**, Isolated DNA was also subjected to 16S rDNA PCR analysis, using universal primers. PCR product was observed for all samples assessed. **C**, Serum from 3 healthy donors was incubated at 30°C for 5 and 10 days, and then analyzed using a 16S rDNA qPCR assay. No significant difference in 16S rDNA was observed between the different time points.

To further assess whether bacterial DNA is present in the supernatant of stored RBC units, a large microparticle pellet was isolated by sequential centrifugation of RBCs (100mL) that had been stored for 42 days under standard blood bank conditions. The pellet contained ~3 billion submicron, calcein-positive vesicles, as determined by flow cytometric analysis. DNA was extracted from the pelleted material and analyzed by standard gel electrophoresis. *S*. *pneumoniae* was used as a positive control, and molecular grade water or molecular grade water passed through the Qiagen DNA extraction protocol were negative controls. Despite the isolation of very large numbers of these vesicles, no genomic DNA was observed by gel electrophoresis (Fig 3A). Similar results were obtained using a phenol-chloroform based DNA extraction protocol. Subsequently, PCR was performed on the pelleted material using the same universal 16S rDNA primers as described by McLaughlin et al. PCR product was observed in pelleted vesicles and both positive and negative controls (Fig 3B). The PCR product from each sample was purified and sequenced by single pass sequencing (Beckman Coulter Genomics) using the same 16S rDNA primers (S1 File). BLAST analysis revealed that 16S sequences in the pelleted material were highly similar to *Pelomonas spp*. Sequences from molecular grade water and water passed through the Qiagen extraction protocol were related to *Bradyrhizobium spp*. and *Propionibacterium spp*., respectively. All three species are known to be common contaminants in laboratory reagents [6]. Of note, both species identified by McLaughlin et al. are also now known to be typical contaminants (*Stenotrophomonas spp*., *Pseudomonas spp*.). As expected, 16S rDNA amplified from *S*. *pneumonia* DNA was identified as *S*. *pneumonia* (raw sequences available in supplemental material).

### 16S rDNA levels are unchanged in human serum incubated for up to 10 days

To further rule out the possibility of viable bacteria in the blood of healthy humans, serum from 3 donors was subjected to 16S rDNA qPCR analysis after incubation for 0, 5, and 10 days. The abundance of 16S rDNA would be expected to increase over time if viable, replicating bacteria were present. Here, we did not find any change in the amount of 16S rDNA over time (ANOVA, p = 0.53) (Fig 3C).

### Flow cytometry, miRNA, and protein analysis indicate isolated structures are RMPs

**Fig 4:**
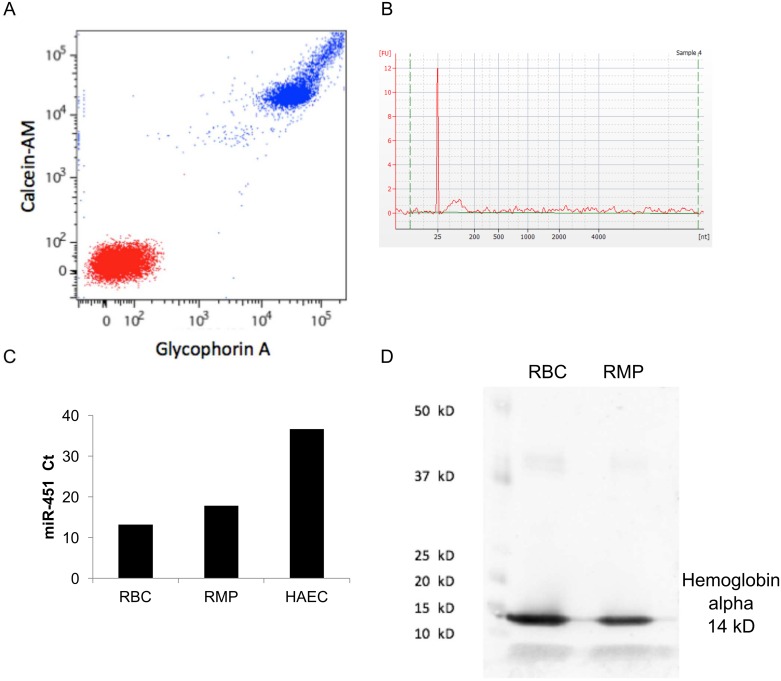
Vesicles isolated from supernatant of RBC storage units are membrane-bound, intact, and contain RBC surface antigen and RBC-specific miRNA. **A**, Unstained vesicles (red, bottom left) or vesicles co-stained with calcein-AM and fluorescent anti-GPA (blue, top right) were analyzed by flow cytometry. >99% of the vesicles were positive for calcein-AM and anti-GPA. **B**, RNA from vesicles was analyzed by Agilent Bioanalyzer. Peak on electropherogram at 25 nt is internal standard and small peak to the right reflects small RNA. This electropherogram is representative of Bioanlyzer data from six different RBC storage units. **C**, Levels (Ct values) of miR-451 were assessed by qRT-PCR in stored RBCs, the RMP pellet, and in human aortic endothelial cells (HAECs, negative control). **D**, Hemoglobin-alpha content of RMP pellet and stored RBCs, as assessed by Western blot. Blot is representative of six different RBC storage units.

Flow cytometric analysis demonstrated that all vesicles in the pelleted material from RBC storage units were positive for glycophorin-A (GPA), a RBC specific surface antigen, and calcein-AM (Fig 4A), suggesting that they were membrane-bound and intact [7]. Calcein-AM is a non-fluorescent, membrane permeable dye that becomes fluorescent and membrane impermeant after it is cleaved by esterases. RNA isolated from the vesicles was characterized using an Agilent Bioanalyzer and revealed that it contained only small RNA (Fig 4B). miR-451, a miRNA known to be highly enriched in RBCs was highly abundant in both RBC and in RMP fractions, as compared to RNA isolated from cultured human aortic endothelial cells (HAECs), which have low levels of miR-451 and were used as a negative control (Fig 4C). High levels of hemoglobin-alpha were detected in protein lysate of the vesicles and RBCs (Fig 4D). Since the RMP pellet was washed extensively with PBS prior to RNA or protein isolation, it is not expected that the miR-451 or hemoglobin-alpha was derived from lysed RBCs in the stored unit.

## Discussion

In summary, unfixed negative-stain EM of material in the extracellular fluid of RBC storage units identified pleomorphic structures that appeared identical to those previously reported to be circulating bacteria. Our results suggest that these pleomorphic structures are RMPs, not viable bacteria. First, genomic DNA was undetectable in the extracellular material, even when very large numbers of vesicles were used for DNA isolation. Second, incubation of serum samples from healthy humans at 30°C for up to 10 days failed to show any increase in bacterial 16S rDNA content. Third, bacterial 16S rDNA sequences amplified by PCR, both in this study and in previous studies, are known to be common contaminants in laboratory reagents [6]. Lastly, isolated vesicles had intact membranes, RBC specific antigen on their surface, and relatively abundant amounts of hemoglobin alpha and miR-451, a miRNA highly expressed in RBCs [8].

The unusual, pleomorphic nature of the vesicles present in unfixed negative-stain EM appeared to largely be due to the vesicles remaining flexible and deformable just prior to grid preparation. Whereas, most vesicles were observed to be ellipsoidal when fixed for negative-stain EM, thin-section TEM, or SEM. However, a small fraction of particles seemed to have a rod-shaped morphology. Why RBC-derived vesicles would assume this seemingly energetically unfavorable morphology is not clear, though it may be related to the unique structural proteins or lipid content responsible for the biconcave shape of RBCs. The cryo-EM studies revealed the presence of “full” and “empty” MPs. We suspect the granular material in the full MPs is hemoglobin, and that the empty MPs are ghost MPs, which others have observed in stored RBC units[9].

While it is accepted that bacteria transiently transmigrate into healthy human circulation, the concept that naturally occurring, viable nanobacteria routinely circulate in blood is not well supported. In general, the existence of nanobacteria is controversial [10], and, whereas multiple groups have provided evidence that nanobacteria-like structures can form spontaneously in human serum, the consensus opinion is that these structures are likely mineraloprotein complexes rather than microbes [11, 12].

## Conclusions

The existence of pleomorphic nanobacteria in healthy human blood has been described elsewhere; however, our study suggests that pleomorphic structures observed in negative-stain electron micrographs of human RBC storage units are RMPs and that 16S rDNA PCR products associated with these RMPs are contaminants, commonly known to be present in laboratory reagents.

## Methods

The Emory University IRB approved all studies and study participants gave written consent prior study participation.

### Isolation of RBC-derived vesicles

Leukocyte-depleted RBC transfusion units were obtained from the blood bank at Emory University Hospital and stored from 0–42 days under standard conditions at 4°C. Blood product samples were obtained through a syringe port using a sterile 18-gauge needle under aseptic conditions. Samples were then prepared using a protocol developed to isolate microparticles (MPs), based on???. RBCs were centrifuged at 1900 x g for 1 minute to pellet cells, the supernatant was transferred to a sterile tube, and centrifuged a second time at 800 x g for 10 minutes to pellet any residual RBCs. The supernatant was then centrifuged at 16,100 x g for 20 minutes to pellet MPs. The MP pellet was re-suspended in molecular grade PBS and either studied immediately or frozen in aliquots at minus 80°C.

### Electron microscopy

#### Negative staining

The Robert P. Apkarian Integrated Electron Microscopy Core, Emory University performed transmission electron microscopy of the RBC vesicles using a standard negative staining protocol. Briefly, 400-mesh carbon coated copper grids were made hydrophilic by glow discharging. A 5 μl droplet of the vesicle isolate, either unfixed or fixed with 2.5% glutaraldehyde, was placed on the grid, after 5 minutes, the grid with the suspension was rinsed by briefly touching the sample side to three drops of distilled water. The excess water on the grid was then removed by blotting the side of the grid on a piece of filter paper. For negative staining, 5 μl of 1% phosphotungstic acid (PTA) was applied onto the grid immediately after water removal, and then removed as described above after 30 seconds. The grid was allowed to completely dry before viewing in the microscope.

#### TEM

For thin-section TEM examination of embedded RBC vesicles, the samples were fixed with 2.5% glutaraldehyde in 0.1 M sodium cacodylate (pH 7.4). Samples were then washed with the same buffer twice and post-fixed with 1% osmium tetroxide and 1.5% potassium ferrocyanide in the same buffer, dehydrated through a graded ethanol series to 100%, and embedded in Eponate 12 resin (Ted Pella Inc., Redding, CA). Ultrathin sections were cut on a Leica UltraCut S ultramicrotome (Leica Microsystems Inc., Buffalo Grove, IL) at 70 nm, and counter-stained with 4% aqueous uranyl acetate and 2% lead citrate. Sections were examined using a 120 kV JEOL JEM-1400 LaB_6_ transmission electron microscope (JEOL, Ltd., Japan) equipped with a Gatan 2k x 2k US1000 CCD camera (Gatan, Inc., Pleasanton, CA).

#### SEM

For SEM examination of RBC vesicles, the samples were fixed with 2.5% glutaraldehyde in 0.1 M sodium cacodylate buffer (pH 7.4). The samples were then placed onto poly-L-lysine coated silicon chips and washed with the same buffer twice before post-fixation with 1% osmium tetroxide in the same buffer and dehydration through a graded ethanol series to 100%. Silicon chips with vesicles were then loaded into a Polaron E3000 critical point drying apparatus to exchange 100% ethanol for liquid CO_2_. Once liquid CO_2_ was brought to its critical point, it was vented slowly. The samples on the silicon chips were coated with 8 nm chromium in a Denton DV-602 Turbo Magnetron Sputter Coater (Denton Vacuum, LLC, Moorestown, NJ) before imaging on the upper-stage of a Topcon DS-150 field emission-scanning electron microscope (FE-SEM).

#### Cryo-electron microscopy and cryo-electron tomography

Purified RBC microparticles (4 μl) were applied to Quantifoil R 2/1 TEM grids and plunge frozen using an FEI Vitrobot Mark III system (FEI, Hillsboro, OR). 10 nm BSA-treated colloidal gold fiducial markers were mixed with the sample prior to freezing. Images were acquired using a JEOL JEM-2200FS field-emission TEM (JEOL Ltd., Japan) operated at 200 kV and equipped with an in-column Omega energy filter (slit width: 20 eV). Montages were collected on a Gatan US4000 4k × 4k CCD camera (Gatan) at 10,000× nominal magnification. Montages were stitched together prior to particle counting and diameter measurements. Diameters were measured using the *imodinfo* command by placing open model points at two ends of the particles. Tilt series were collected using the SerialEM software package [13] from -62° to 62° at 2° increments, with a defocus of -6 μm and a total dose of ~135 e^-^/Å^2^. Tilt series were recorded on a Direct Electron DE-20 (Direct Electron, LP, CA) at 12 frames per second and a nominal magnification of 10,000× (pixel size 6.14 Å) [14]. Frames were motion corrected using scripts provided by the vendor (DE). The images were CTF-corrected by phase inversion and the three-dimensional reconstructions were calculated via the weighted-back projection algorithm using IMOD software [15]. The reconstructed volumes (binned by 2) were further denoised using nonlinear anisotropic diffusion as implemented in IMOD.

### Microbial DNA analysis

DNA was isolated from microparticle pellets, molecular grade water, and a colony of *Streptococcus pneumoniae* using a QIAamp DNA Mini Kit according to the manufacturer’s specified protocol for bacteria (Qiagen). As an alternative, DNA was extracted using a conventional phenol-chlorophorm extraction protocol[16]. Standard agarose gel electrophoresis was carried out on a 1% agarose accompanied by a 1kb DNA ladder (NEB). The same universal 16S PCR primers used by McLaughlin et al. were utilized to amplify 16S rDNA: BSF8/20 (5′-AGAGTTTGATCCTGGCTCAG-3′) and the reverse primer BSR1541/20 (5′-AAGGAGGTGATCCAGCCGCA-3′)[2]. PCRs were performed in 20 μl reaction volumes with 1X Q5 High Fidelity Master Mix (New England Biolabs), 1 μM forward and reverse primer, and 6 ul of DNA template. Reactions were carried out on a Biorad C1000 Touch Thermocycler with the following conditions: 98°C x 10 minutes, (98°C x 10 seconds, 70°C x 30 seconds, 72°C x 30 seconds) x 38 cycles, 72°C x 2 minutes. PCR products were purified using a Qiaquick PCR Purification Kit per the manufacturer’s protocol and sent for commercial first pass sequencing (Beckman Coulter) using the BSF8/20 and BSR1541/20 primers.

#### 16S rDNA qPCR of human serum

Under aseptic conditions, blood was collected into serum vacutainers (BD) from 3 healthy donors (no medications). Cells were removed by centrifugation (3,000 x g for 7 minutes) and serum was incubated for 0, 5, or 10 days at 30°C. DNA was extracted from 100 ul of serum as described above and used as a template for qPCR. Universal 16S primers developed previously for 16S rDNA qPCR were utilized: EUBAC-F (5′-TCCTACGGGAGGCAGCAGT-3′) and EUBAC-R (5′-GGACTACCAGGGTATCTAATCCTGTT-3′)[17]. These primers were selected for qPCR because the amplicon size was more appropriate for qPCR studies than the primers used by McLaughlin et al. Reactions were performed in 20 μl volumes with 1X Quantitect SYBR Green Master Mix, 300nM forward and reverse primers, and 8.8 μl DNA. Thermocycler conditions were: 95C x 10 minutes, (95°C x 15 seconds, 60°C x 1 minute) x 40 cycles. All reactions were performed in triplicate. Relative expression was calculated as 2^-dCt^ and converted to fold-change by normalizing to the mean expression at day 0. Data was analyzed using a one-way ANOVA with the assistance of Prism (Graphpad).

### Flow cytometry

Isolated particles were stained with 10 μM calcein-AM (Life Technologies) and anti-CD235a (GPA) fluorescently labeled antibody (PE/Cy7; BioLegend), incubated at room temperature for 20 minutes, and analyzed on a BD LSR flow cytometer (BD Biosciences). The concentration of RMPs in a sample was calculated by ratiometric comparison after adding a known concentration of Flow-Check Fluoroshperes (Beckman).

### miRNA analysis

Total RNA was isolated using a miRNeasy Mini Kit according to the manufacturer’s protocol (Qiagen) and analyzed on an Agilent Bioanalyzer with the RNA 6000 Pico Kit by standard protocol. qRT-PCR analysis of miR-451 was performed using Qiagen products (miScript II RT Kit, miScript Primer assay) and analyzer on a StepOne Real-Time PCR System (ThermoFisher).

### Western blot

Particles were lysed in RIPA buffer, exposed to brief sonication, and protein was quantified using the BCA assay. 0.3 μg of protein was separated by gel electrophoresis, transferred to a nitrocellulose membrane, and probed using an anti-hemoglobin-alpha antibody (Santa Cruz Biotechnology; sc-21005).

## Supporting Information

S1 File PCR product raw sequences. PCR products were purified as described and first pass sequencing was performed using the BSF8/20 and BSR1541/20 primers.
(TXT)

## References

[1] Hopewell S, Omar O, Hyde C, Yu LM, Doree C, Murphy MF. A systematic review of the effect of red blood cell transfusion on mortality: evidence from large-scale observational studies published between 2006 and 2010. BMJ open. 2013;3(5). 10.1136/bmjopen-2012-002154 23645909 PMC3646177

[2] McLaughlin RW, Vali H, Lau PC, Palfree RG, De Ciccio A, Sirois M, et al Are there naturally occurring pleomorphic bacteria in the blood of healthy humans? Journal of clinical microbiology. 2002;40(12):4771–5. 10.1128/JCM.40.12.4771-4775.2002 12454193 PMC154583

[3] Szymanski M, Petric M, Saunders FE, Tellier R. Mycoplasma pneumoniae pericarditis demonstrated by polymerase chain reaction and electron microscopy. Clinical infectious diseases: an official publication of the Infectious Diseases Society of America. 2002;34(1):E16–7. 10.1086/338158 . 11731968

[4] Rubin O, Canellini G, Delobel J, Lion N, Tissot JD. Red blood cell microparticles: clinical relevance. Transfusion medicine and hemotherapy: offizielles Organ der Deutschen Gesellschaft fur Transfusionsmedizin und Immunhamatologie. 2012;39(5):342–7. 10.1159/000342228 23801926 PMC3678264

[5] Dinkla S, Brock R, Joosten I, Bosman GJ. Gateway to understanding microparticles: standardized isolation and identification of plasma membrane-derived vesicles. Nanomedicine. 2013;8(10):1657–68. 10.2217/nnm.13.149 . 24074388

[6] Salter SJ, Cox MJ, Turek EM, Calus ST, Cookson WO, Moffatt MF, et al Reagent and laboratory contamination can critically impact sequence-based microbiome analyses. BMC biology. 2014;12:87 10.1186/s12915-014-0087-z 25387460 PMC4228153

[7] Gray WG, Mitchell AJ, Searles CS. An accurate, precise method for general labeling of extracellular vesicles. MethodsX. 2015;2:360–7. 10.1016/j.mex.2015.08.002 26543819 PMC4589801

[8] Azzouzi I, Moest H, Wollscheid B, Schmugge M, Eekels JJ, Speer O. Deep sequencing and proteomic analysis of the microRNA-induced silencing complex in human red blood cells. Experimental hematology. 2015;43(5):382–92. 10.1016/j.exphem.2015.01.007 . 25681748

[9] Salzer U, Zhu R, Luten M, Isobe H, Pastushenko V, Perkmann T, et al Vesicles generated during storage of red cells are rich in the lipid raft marker stomatin. Transfusion. 2008;48(3):451–62. 10.1111/j.1537-2995.2007.01549.x . 18067507

[10] Young JD, Martel J. The rise and fall of nanobacteria. Scientific American. 2010;302(1):52–9. 10.1038/scientificamerican0110-52 . 20063636

[11] Martel J, Young JD. Purported nanobacteria in human blood as calcium carbonate nanoparticles. Proceedings of the National Academy of Sciences of the United States of America. 2008;105(14):5549–54. 10.1073/pnas.0711744105 18385376 PMC2291092

[12] Raoult D, Drancourt M, Azza S, Nappez C, Guieu R, Rolain JM, et al Nanobacteria are mineralo fetuin complexes. PLOS pathogens. 2008;4(2):e41 10.1371/journal.ppat.0040041 18282102 PMC2242841

[13] Mastronarde DN. Automated electron microscope tomography using robust prediction of specimen movements. J Struct Biol. 2005;152(1):36–51. 10.1016/j.jsb.2005.07.007 . 16182563

[14] Yi H, Strauss JD, Ke Z, Alonas E, Dillard RS, Hampton CM, et al Native immunogold labeling of cell surface proteins and viral glycoproteins for cryo-electron microscopy and cryo-electron tomography applications. J Histochem Cytochem. 2015;63(10):780–92. 10.1369/0022155415593323 . 26069287 PMC4823802

[15] Kremer JR, Mastronarde DN, McIntosh JR. Computer visualization of three-dimensional image data using IMOD. J Struct Biol. 1996;116(1):71–6. 10.1006/jsbi.1996.0013 . 8742726

[16] Bergallo M, Costa C, Gribaudo G, Tarallo S, Baro S, Negro Ponzi A, et al Evaluation of six methods for extraction and purification of viral DNA from urine and serum samples. The new microbiologica. 2006;29(2):111–9. . 16841551

[17] Dinakaran V, Rathinavel A, Pushpanathan M, Sivakumar R, Gunasekaran P, Rajendhran J. Elevated levels of circulating DNA in cardiovascular disease patients: metagenomic profiling of microbiome in the circulation. PLOS ONE. 2014;9(8):e105221 10.1371/journal.pone.0105221 25133738 PMC4136842

